# Political Impediments to Aging in Place: The Example of Informal Caregiving Policy

**DOI:** 10.1093/geroni/igaa057.2369

**Published:** 2020-12-16

**Authors:** Jacqueline Chattopadhyay

**Affiliations:** UNC Charlotte, Charlotte, North Carolina, United States

## Abstract

Most Americans prefer to “age-in-place” as long as possible, but to do so often need overlapping resources—one of which is help from “formal” or “informal” caregivers (family and friends). Family and friends often want to provide care for as long as safely possible. However, informal caregiving can pose financial and physical risks to the caregiver that—as many scholars have noted—public policy in the U.S. does relatively little to mitigate. This policy shortfall also hurts care recipients since the risks that informal caregivers face can prematurely curtail their ability to provide care. Why does policy in the U.S. not better support informal caregivers? By synthesizing family caregiving research and political science research that has addressed long-term care, this paper surveys nine factors in the political system that may help answer this question. Four emanate from policy history. Three concern the mass public. Two vary at the policy level.

